# A blood-based epigenetic clock for intrinsic capacity predicts mortality and is associated with clinical, immunological and lifestyle factors

**DOI:** 10.1038/s43587-025-00883-5

**Published:** 2025-06-04

**Authors:** Matías Fuentealba, Laure Rouch, Sophie Guyonnet, Jean-Marc Lemaitre, Philipe de Souto Barreto, Bruno Vellas, Sandrine Andrieu, David Furman

**Affiliations:** 1 Buck AI Platform, Buck Institute for Research on Aging, Novato, CA USA; 2 IHU HealthAge, Toulouse, France; 3 CERPOP UMR 1295, Inserm, Université Paul Sabatier, Toulouse, France; 4 INSERM IRMB UMR1183, Hôpital Saint Eloi, Université de Montpellier, Montpellier, France; 5 Department of Clinical Epidemiology and Public Health, Toulouse University Hospital, Toulouse, France; 6 Stanford 1000 Immunomes Project, Stanford School of Medicine, Stanford, CA USA; 7 Edifice Health Inc., San Mateo, CA USA

**Keywords:** Ageing, Computational models, Predictive markers, Ageing

## Abstract

Age-related decline in intrinsic capacity (IC), defined as the sum of an individual’s physical and mental capacities, is a cornerstone for promoting healthy aging by prioritizing maintenance of function over disease treatment. However, assessing IC is resource-intensive, and the molecular and cellular bases of its decline are poorly understood. Here we used the INSPIRE-T cohort (1,014 individuals aged 20–102 years) to construct the IC clock, a DNA methylation-based predictor of IC, trained on the clinical evaluation of cognition, locomotion, psychological well-being, sensory abilities and vitality. In the Framingham Heart Study, DNA methylation IC outperforms first-generation and second-generation epigenetic clocks in predicting all-cause mortality, and it is strongly associated with changes in molecular and cellular immune and inflammatory biomarkers, functional and clinical endpoints, health risk factors and lifestyle choices. These findings establish the IC clock as a validated tool bridging molecular readouts of aging and clinical assessments of IC.

## Abstract (web summary)

Fuentealba and colleagues build and validate a DNA methylation clock of intrinsic capacity (IC) to bridge resource-intensive clinical assessments of IC with molecular readouts of aging, highlighting strong links to inflammation, clinical health markers and lifestyle factors.

## Main

In 2015, the World Health Organization (WHO) introduced the concept of intrinsic capacity (IC), defined as the sum of all physical and mental capacities that an individual can draw on at any point in their life^1^. This concept promotes healthy aging by shifting the healthcare focus from treating acute illnesses toward measuring and preserving functional ability^1–3^. Although IC varies between individuals, it peaks in early adulthood, declines after midlife and can be improved at any age through lifestyle^4–9^.

The International Classification of Diseases, 11th Revision, recently added ‘aging-associated decline in IC’ under code MG2A^10^, standardizing the clinical use of IC globally as a metric of functional aging. Since the inception of IC, many studies have developed IC scores and demonstrated its association with health-related factors^11^, including linking low IC to higher comorbidity, frailty, difficulties in activities of daily living and increased falls^12^.

Despite the advantages of using IC to assess functional ability, current methods to quantify it require equipment and trained personnel, and the molecular and cellular mechanisms underlying its age‑associated decline are still poorly understood. To address these, we collected DNA methylation (DNAm) data from participants in the INSPIRE Translational (INSPIRE-T) cohort to construct an epigenetic predictor of IC (IC clock). Then, we applied the IC clock to the Framingham Heart Study (FHS) to evaluate associations between DNAm IC and mortality, clinical markers of health and lifestyle, and explore the molecular and cellular mechanisms of IC using transcriptomics data and cell composition changes.

## Results

### IC declines with age

**Fig. 1:**
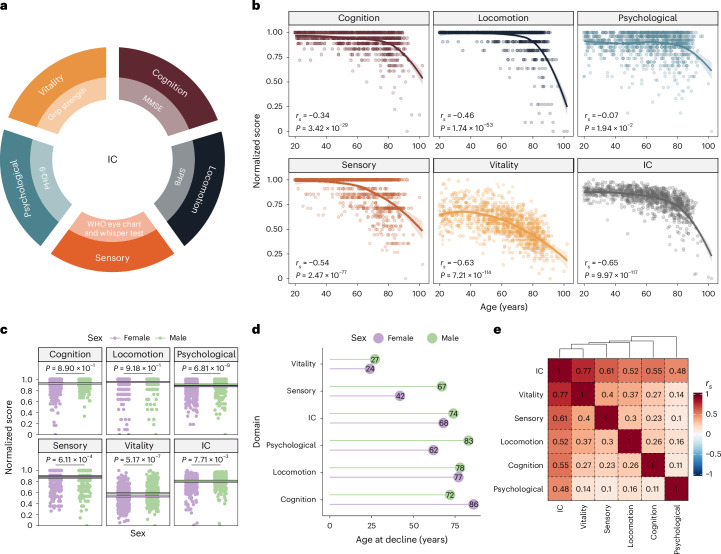
Age-related and sex-related changes in IC. **a**, Domains of IC and clinical assessment tools used to derive the IC score. **b**, Correlation between the scores in each domain (and overall) and chronological age. Because of the nonlinear relationship with age, correlation values were calculated using Spearman’s rank correlation coefficients. *P* values were approximated via two-sided *t*-distribution. We estimated the regression line using locally estimated scatterplot smoothing. The shaded regions represent the 95% confidence intervals (CIs) around the smoothed line. **c**, Average scores for each IC domain in male (green) and female (purple) participants. The lines indicate the mean value for each sex in each domain. We calculated the two-sided *P* values for the mean differences using a Wilcoxon’s rank-sum test. **d**, Estimated age of decline in each domain obtained from a continuous two-phase model regression analysis. **e**, Spearman’s rank correlation coefficients between each IC domain’s score. MMSE, Mini-Mental State Examination; SPPB, Short Physical Performance Battery; PHQ-9, Patient Health Questionnaire-9.

Using clinical assessment tools, we developed an IC score that represents combined age-related decline in five domains: cognition; locomotion; sensory (vision and hearing); psychological; and vitality (Methods) (Fig. 1a). IC scores ranged from 0 to 1, with 1 indicating the best possible health outcome and 0 representing the worst. We examined the correlations between chronological age and each domain. All IC domains correlated negatively with age, with the strongest correlation observed for overall IC (*r*_*s*_ = −0.65, *P* = 9.97 × 10^−117^) (Fig. 1b) and the weakest with the psychological domain (*r*_*s*_ = −0.07, *P* = 1.94 × 10^−2^).

**Extended Data Fig. 1:**
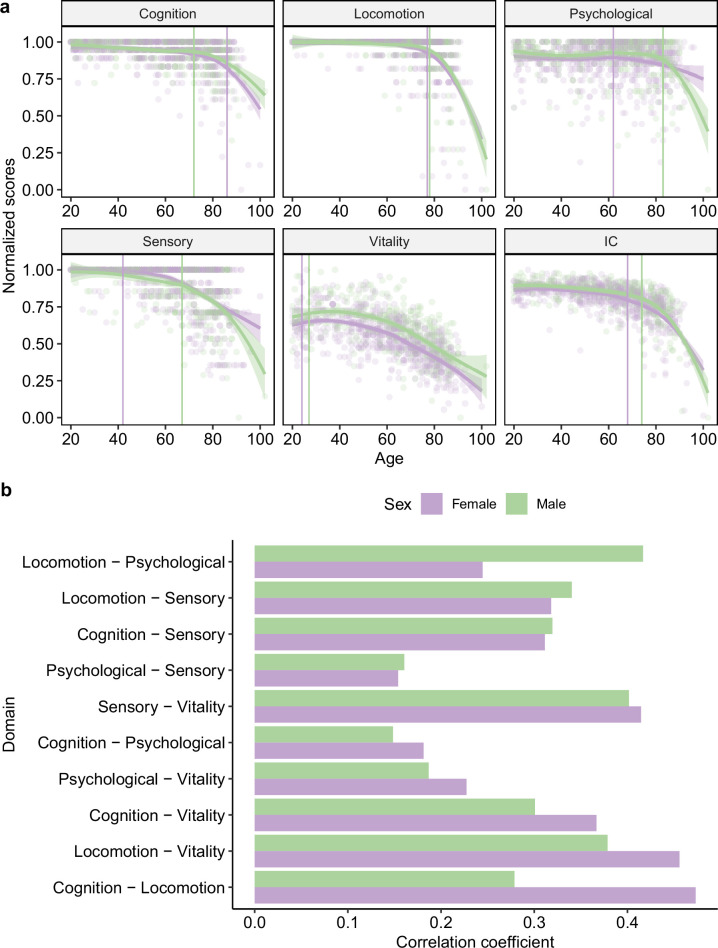
Sex-specific age trajectories of intrinsic capacity domains. **a**. Panels illustrate the locally estimated scatterplot smoothing (LOESS) curves for the IC domains stratified by sex (n = 973 individuals). Shaded regions represent the 95% confidence intervals around the smoothed line. Vertical lines indicate the age at which IC decline begins in males (green) and females (purple) in the INSPIRE-T cohort. **b**. Pairwise Pearson’s correlations between IC domains in males and females. Domains were ordered by the difference observed between males and females.

Given the well-established differences in health span and lifespan between sexes, we investigated sex differences in the levels and age at decline of the IC domains. We found that males had higher scores in the psychological and vitality domains (*P* = 6.81 × 10^−9^ and 5.17 × 10^−7^, respectively), while females had higher scores in the sensory domain (*P* = 6.11 × 10^−4^) (Fig. 1c and Extended Data Fig. 1a). Cognition and locomotion did not show statistically significant differences between sexes (*P* > 0.05). Using a continuous two-phase model regression analysis, we found that female participants exhibited an earlier sensory decline (42 versus 67 years) (Fig. 1d), whereas male participants exhibited earlier cognitive decline (72 versus 86 years), suggesting that females tended to maintain cognitive resilience for longer.

We next assessed the contribution of each domain by calculating their correlation with overall IC. The overall IC score had a stronger positive correlation with each domain (*r*_*s*_ ranging from 0.48 to 0.77) than the correlations between domains, confirming the integrative nature of the IC score (Fig. 1e). The sensory and vitality domains showed the highest inter-domain correlation (*r*_*s*_ = 0.4), while the psychological and sensory domains had the lowest (*r*_*s*_ = 0.1). We also analyzed the correlation between domains for each sex and observed that in male individuals, there was a higher correlation between locomotion and the psychological or sensory domains, whereas in female individuals, locomotion was correlated with vitality and cognition (Extended Data Fig. 1b). This result might reflect sex-specific effects on other domains when locomotion is impaired.

### DNAm-based predictor of IC

**Fig. 2:**
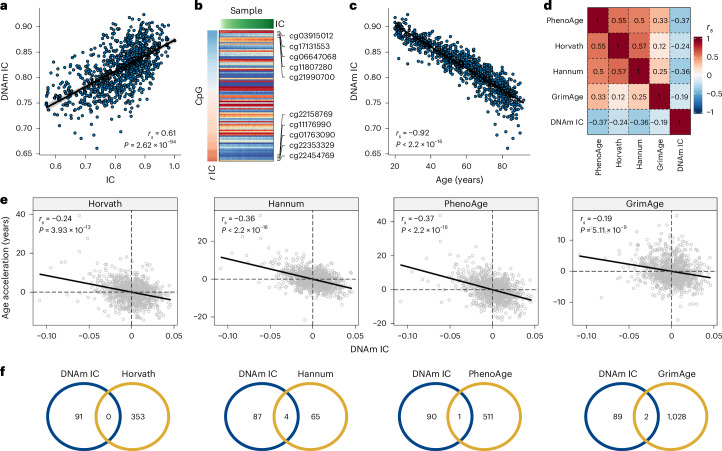
Model selection and correlation with epigenetic clocks. **a**, Spearman’s rank correlation between IC and the DNAm-based estimate of IC (DNAm IC). **b**, DNAm levels of the 91 CpGs in the best predictive model. Samples were sorted according to IC values; CpGs were sorted according to their correlation with IC. **c**, Spearman’s rank correlation between DNAm IC and chronological age. **d**, Spearman’s rank correlations between age acceleration and epigenetic clocks (that is, age-adjusted epigenetic age) including DNAm IC. **e**, Scatter plots representing the relationship between the age acceleration of DNAm IC and the epigenetic clocks Horvath, Hannum, PhenoAge and GrimAge. Spearman’s rank correlation coefficients and *P* values for the association are displayed in the top left corner of each panel. **f**, Overlap between CpGs included in the IC clock (blue circles) and epigenetic clocks (yellow circles).

**Extended Data Fig. 2:**
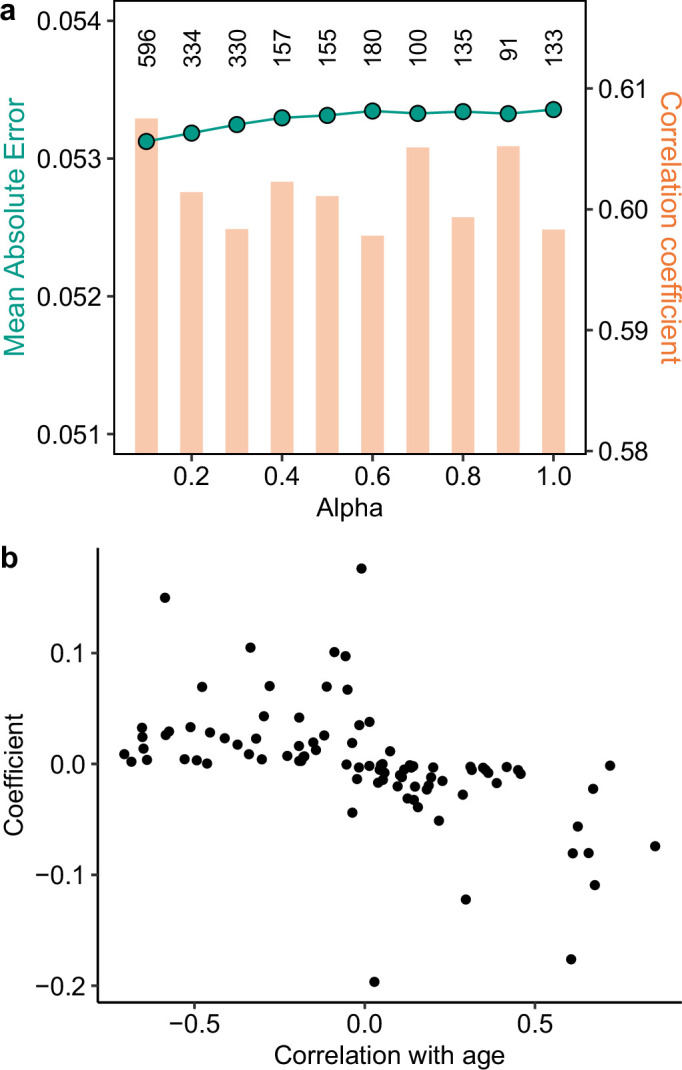
Model performance and age influence in IC clock features. **a**. Elastic net model performance metrics at different alpha parameters ranging from 0.1 to 1. Orange bars indicate the correlation between actual and predicted IC values. Green dots show the mean absolute error, and the numbers above indicating the number of CpGs included in each model. **b**. Dependency of feature importance and age correlation. Relationship between the correlation of CpG beta values with age (x-axis) and the model’s coefficients for the 91 CpGs included in the IC clock.

We used DNAm data (Infinium EPIC array) from 933 INSPIRE-T participants to predict IC. We built the predictive model using elastic net regression and tenfold cross-validation^13^. We ranked models with different elastic net mixing parameters (alpha) based on the correlation between observed and cross-validated predicted values, the model’s error and the number of cytosine-phosphate-guanine (CpG) sites used (Extended Data Fig. 2a). The model with the best ranking across all three metrics (highest correlation, lowest error and fewer CpGs) resulted in a correlation of 0.61 between IC and predicted values based on DNAm (Fig. 2a) and included 91 CpGs (Fig. 2b and Supplementary Table 1). The IC estimations based on the DNAm data showed a strong correlation with age (*r*_*s*_ = −0.92; Fig. 2c), although the CpGs with the highest coefficients displayed nearly zero correlation with chronological age (Extended Data Fig. 2b).

We compared the IC clock to first-generation and second-generation clocks (Horvath, Hannum, PhenoAge and GrimAge). We found a negative correlation between DNAm IC and the epigenetic clocks, with PhenoAge showing the strongest correlation (*r*_*s*_ = −0.37, *P* < 2.2 × 10^−16^), followed by the Hannum clock (*r*_*s*_ = −0.36, *P* < 2.2 × 10^−16^) (Fig. 2d,e). Furthermore, the absolute magnitude of the correlation was higher between epigenetic clocks compared to DNAm IC (absolute mean *r*_*s*_ = 0.38 versus 0.29), suggesting that the IC clock captured a distinct aspect of the biology of aging. In line with this finding, no major overlaps between the CpG sites included in the epigenetic clocks and DNAm IC were found (Fig. 2f).

**Extended Data Fig. 3:**
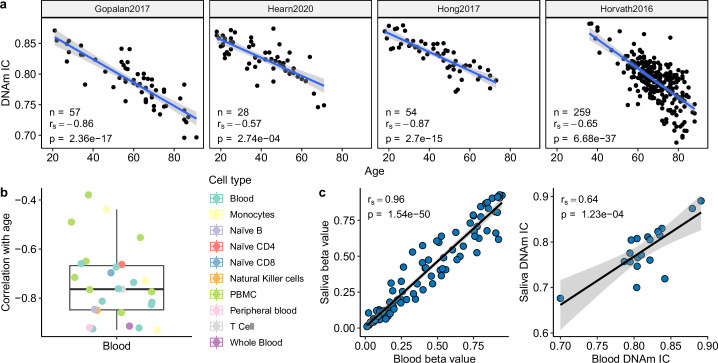
Age-associated decline in DNAm IC estimated in external datasets of blood and saliva. **a**. Evaluation of DNAm IC in saliva samples across four datasets. Correlations and p-values between chronological age and DNAm IC were calculated using the Spearman’s method. **b**. Age-associated decline in DNAm IC estimated from blood samples. Evaluation of DNAm IC in blood or blood-related cell-type samples across 27 datasets. **c**. Comparison of blood and saliva DNAm IC estimates. The scatterplot on the left compares the beta values of the 91 CpG sites used in the DNAm IC model, while the scatterplot on the right shows the resulting DNAm IC values derived from blood and saliva from the same individuals (n = 19 samples). Shaded region around the fitted regression represents the 95% confidence intervals.

We also tested whether DNAm IC could be calculated using epigenetics from saliva. In four datasets, DNAm IC displayed a strong age-related decline (mean *r*_*s*_ = −0.74) (Extended Data Fig. 3a), which was equivalent to blood-derived predictions in 27 external datasets (mean *r*_*s*_ = −0.74) (Extended Data Fig. 3b). In addition, we used DNAm data from the blood and saliva samples of 19 patients (aged 13–73 years) to directly evaluate the degree of similarity. The methylation levels of the 91 CpGs in the DNAm IC model were highly correlated (mean *r*_*s*_ = 0.96, *P* = 1.54 × 10^−50^), as were the estimations of DNAm IC from blood and saliva (*r*_*s*_ = 0.64, *P* = 1.23 × 10^−4^) (Extended Data Fig. 3c). These findings highlight the potential of saliva as a noninvasive alternative to blood for calculating DNAm IC.

### DNAm IC is associated with alterations in the immune response

**Fig. 3:**
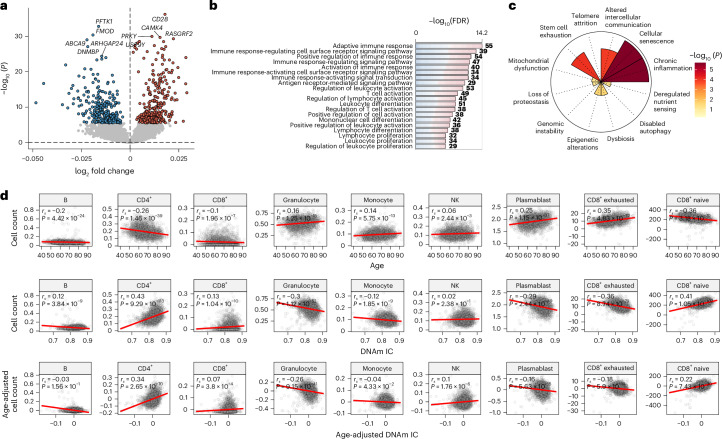
Age-independent gene expression and immune cell frequency correlates of DNAm IC. **a**, Genes whose expression was significantly associated with age-independent changes in DNAm IC (FDR < 0.05, colored dots). The labels indicate the top five genes with the largest changes in each direction after adjustment for multiple testing. Two-sided *P* values were calculated from the *t*-statistics obtained from the linear regression. **b**, Top 20 biological processes enriched (FDR < 0.05) in genes significantly associated with DNAm IC. **c**, Enrichment for the gene sets of the hallmarks of aging. Nominal *P* values were calculated using a one-signed gene set enrichment analysis. **d**, Cell frequency changes with age and DNAm IC. Top, relationship between the DNAm-based cell count estimates and chronological age. Middle and bottom, relationship between DNAm IC and cell count, adjusted or unadjusted according to chronological age. The red lines indicate the linear regression between the variables. The correlations were calculated using Spearman’s rank correlation coefficients; nominal *P* values were derived from the two-sided test of the correlation coefficient (*t*-distribution approximation).

To better understand the molecular components and biological processes associated with DNAm IC, we calculated the IC clock in the FHS and performed differential expression analysis using age-adjusted DNAm IC as the outcome. We found that the expression of 578 genes (286 upregulated and 292 downregulated) was significantly associated with changes in DNAm IC (Fig. 3a and Supplementary Table 2). Among the top genes, higher DNAm IC was strongly associated with a significant increase in expression of *CD28* (false discovery rate (FDR) = 1.07 × 10^−32^), a surface molecule highly expressed in CD4^+^ and CD8^+^ T cells whose loss of expression is a hallmark of immunosenescence. In contrast, poor IC clock levels tracked with elevated expression of *CDK14*/*PFTK1* (FDR = 2.77 × 10^−29^), a regulator of the Wnt signal transduction pathway, a proinflammatory mediator associated with Parkinson’s disease and several cancers, and sensitive to changes in diet^14,15^.

**Extended Data Fig. 4:**
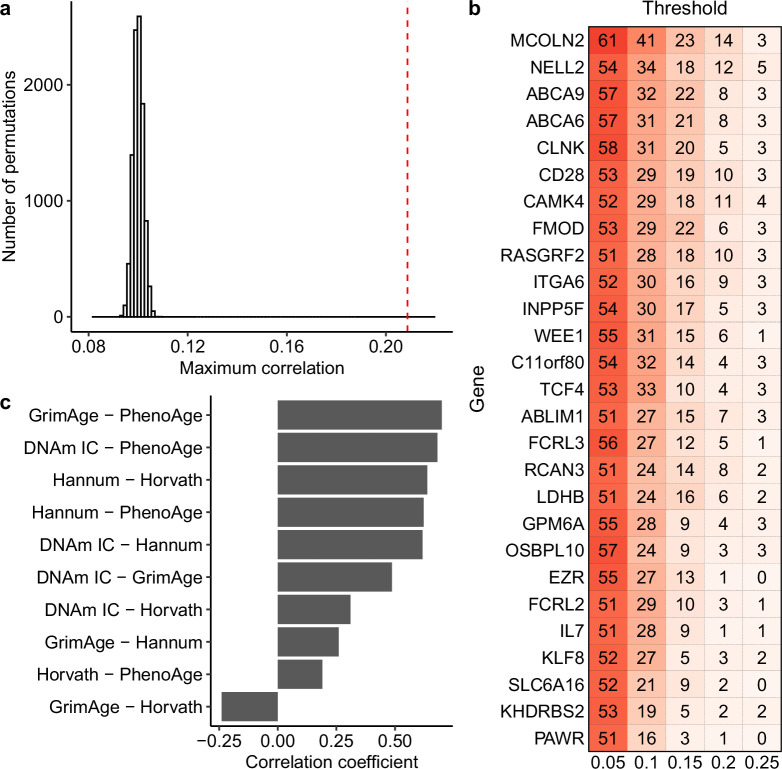
Validation and comparison of gene expression signature of DNAm IC and epigenetic clocks. **a**. Distribution of the absolute maximum correlation between the model’s CpG beta values and the expression of 578 random genes, repeated 10,000 times. The dotted red line indicates the correlation between the model’s CpG beta values and the expression of the 578 genes in the DNAm IC signature. **b**. Genes associated with DNAm CpGs. Number of CpGs with a correlation above different thresholds (x-axis). The top genes with the most correlated CpGs across these correlation thresholds are displayed. **c**. Comparison of gene expression signatures of epigenetic clocks and DNAm IC. Spearman’s Correlation of gene expression changes associated with DNAm IC and those linked to epigenetic clocks (Hannum, Horvath, PhenoAge, and GrimAge).

To confirm the relevance of this signature, we analyzed the relationship between the expression of the 578 genes associated with DNAm IC and the methylation of the 91 CpGs in the IC clock. CpGs in the IC clock showed a correlation of 0.21, with the expression of at least one significantly associated gene, whereas an average correlation of 0.1 is expected by chance (permutation *P* < 0.0001) (Extended Data Fig. 4a). This analysis confirms a strong connection between the IC clock and the identified gene expression signature of DNAm IC.

We also identified which genes showed correlation with the levels of multiple CpGs and found that the expression of *MCOLN2* correlated with the methylation of 61 of 91 CpGs (67%) (Extended Data Fig. 4b and Supplementary Table 3). Lower expression of *MCOLN2* was associated with higher DNAm IC. Interestingly, studies found that MCOLN2 promotes virus entry and infection^16^. Also, *CD28* displayed a correlation with the methylation of more than half (53 of 91 CpGs) of the CpGs in the IC clock. We also examined the similarity between the gene expression signatures associated with epigenetic clocks and DNAm IC (Extended Data Fig. 4c). DNAm IC showed a moderate correlation with PhenoAge and Hannum (*r*_*s*_ = 0.68 and 0.62, respectively) but lower correlation with GrimAge and Horvath (*r*_*s*_ = 0.49 and 0.31, respectively).

To further investigate the gene expression signature of DNAm IC, we performed Gene Ontology (GO) enrichment analysis to identify associated biological processes. Genes associated with age-adjusted DNAm IC were mostly involved in the immune response, particularly T cell activation (Fig. 3b and Supplementary Table 4), which is consistent with the observation that the T cell costimulatory molecule *CD28* was among the top correlated genes. To validate the associations between the IC expression signature and immune system or inflammatory processes, we compiled gene sets for each aging hallmark by leveraging large language models and 36 million journal abstracts from PubMed (Methods). We found that the IC expression signature was strongly enriched in genes involved in cellular senescence and chronic inflammation (Fig. 3c).

We used the Houseman’s method to estimate blood cell counts from epigenetic data and examine the relationship between DNAm IC and cell populations (Fig. 3d). While CD4^+^ T cell frequency decreased significantly with age (*r*_*s*_ = −0.26, *P* = 1.46 × 10^−39^), higher DNAm IC was associated with a greater number of CD4^+^ T cells (*r*_*s*_ = 0.34, *P* = 2.65 × 10^−70^). Similarly, CD8^+^ naive cell numbers decreased with age (*r*_*s*_ = −0.36, *P* = 3.18 × 10^−79^) and increased in individuals with high DNAm IC (*r*_*s*_ = 0.22, *P* = 7.43 × 10^−30^). Unsurprisingly, a negative correlation was found between the number of CD8^+^ exhausted and cytotoxic cells (defined as CD28^−^ and CD45RA^−^) with DNAm IC (*r*_*s*_ = −0.18, *P* = 5.9 × 10^−19^), which could explain why individuals with higher DNAm IC have higher levels of *CD28* expression (‘Discussion’). Overall, these findings indicate that the IC clock detects aspects of immunosenescence in the blood that are associated with functional immune aging changes.

**Extended Data Fig. 5:**
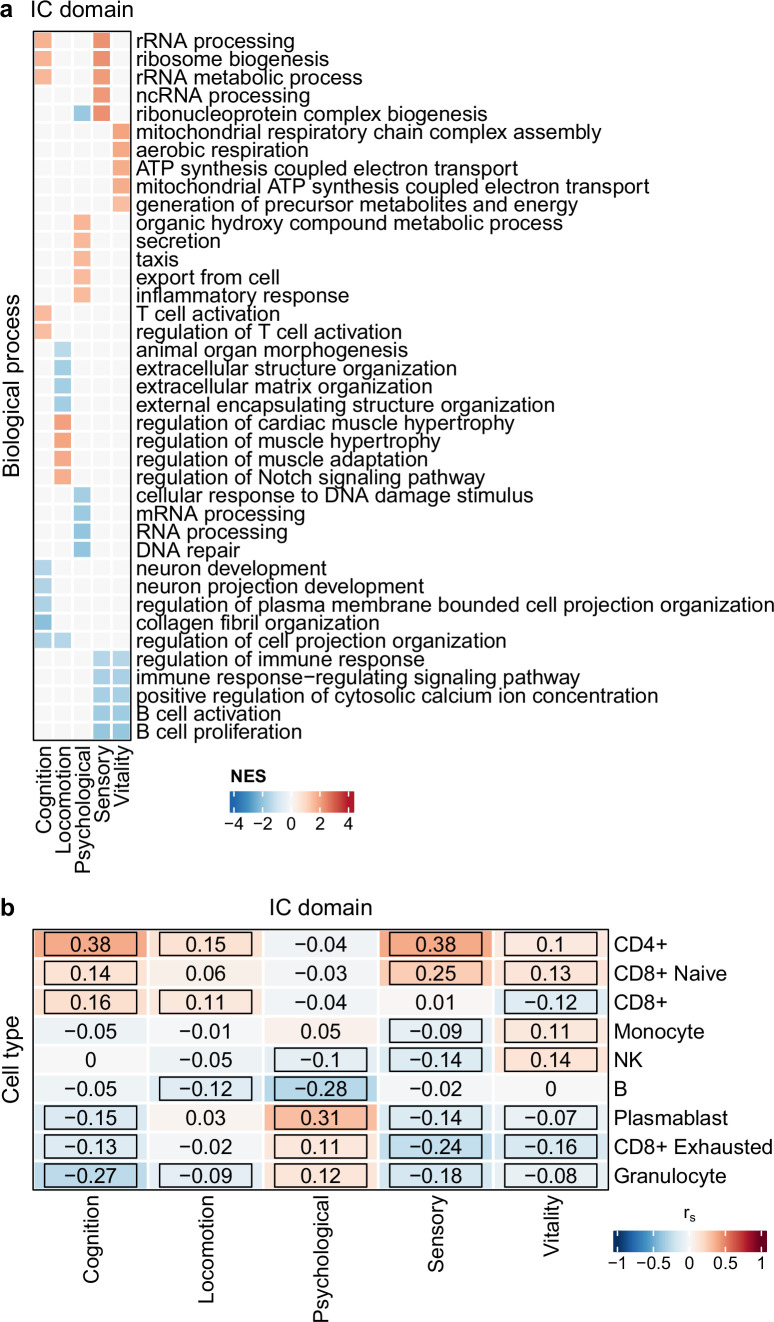
Top biological processes enriched in transcriptional changes associated with DNAm IC domains. **a**. Normalized enrichment scores from a gene set enrichment analysis of the gene expression signatures associated with IC scores predicted from DNA methylation data. The heatmap includes the 5 up- and down-regulated processes associated with each IC domain with the lowest p-values. The color scale represents the Normalized Enrichment Scores (NES). **b**. Correlation between cell frequency changes and DNAm IC domains. Spearman’s correlation between DNAm IC domains scores and cell count (both adjusted by chronological age). Squared numbers indicate correlations with FDR < 0.05.

Next, we aimed to identify molecular correlates and distinguish the potential mechanisms underlying the different domains of IC. We generated DNAm-based predictors for each IC domain in the INSPIRE-T cohort and evaluated the transcriptomic and cell composition changes associated in the FHS cohort. Higher vitality scores were associated with upregulation of mitochondrial electron transport chain genes (FDR = 8.09 × 10^−3^), whereas higher locomotion scores were linked to increased expression of genes regulating cardiac muscle adaptation and the Notch signaling pathway (FDR = 0.01; Extended Data Fig. 5a and Supplementary Table 5). Interestingly, higher cognitive scores were associated with lower expression of pathways involved in neuron development and projection (FDR = 5.19 × 10^−4^), whereas individuals with higher psychological scores showed downregulation of the DNA repair (FDR = 7.75 × 10^−8^). Higher sensory scores were associated with upregulation of ribosome biogenesis and downregulation of immune response pathways (FDR = 1.67 × 10^−8^). In a correlation analysis between changes in cell composition and predicted IC scores, higher cognitive scores were associated with fewer granulocytes (*r*_*s*_ = −0.27) and more CD4^+^ T cells (*r*_*s*_ = 0.38), whereas higher psychological scores correlated with fewer B cells (*r*_*s*_ = −0.28) and more plasmablasts (*r*_*s*_ = 0.31) (Extended Data Fig. 5b). Additionally, higher sensory scores were associated with increased CD4^+^ and CD8^+^ naive T cells (*r*_*s*_ = 0.38 and 0.25, respectively) and fewer CD8^+^ cytotoxic T cells (*r*_*s*_ = −0.24). Vitality and locomotion showed small correlations with cell counts (|*r*_*s*_| < 0.17). These results link genomic instability, mitochondrial dysfunction and loss of proteostasis to the function of specific IC domains.

### DNAm IC is linked to mortality, health markers and lifestyle

**Fig. 4:**
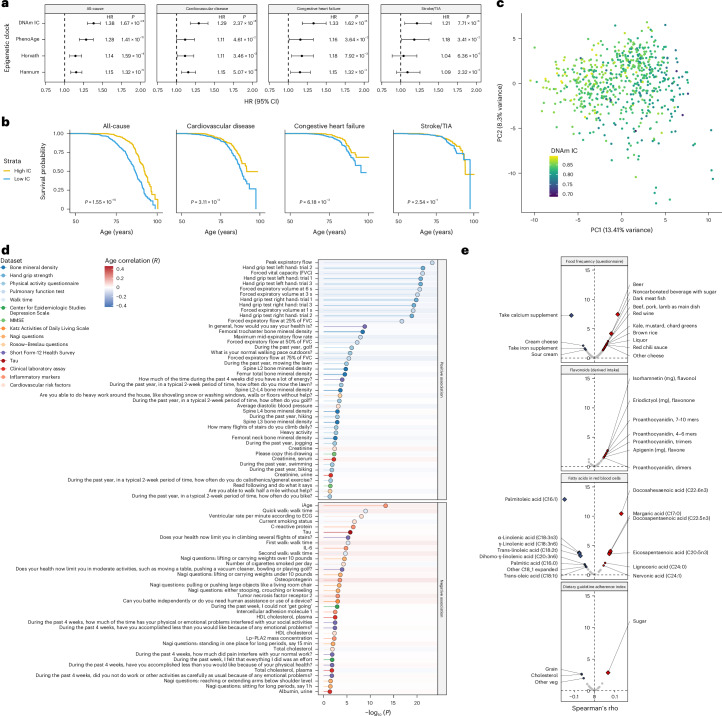
DNAm IC predicts mortality and is associated with functional, clinical and lifestyle factors. **a**, Forest plots summarizing the results from the Cox proportional hazards models adjusted according to chronological age and sex for DNAm IC and first-generation and second-generation epigenetic clocks. The HR was calculated for a one-unit increase in s.d. units of age acceleration. The error bars represent the 95% CIs for the HR estimate from the Cox proportional hazards model. Nominal *P* values for the predictor variable were derived from two-sided Wald tests. **b**, Kaplan–Meier survival estimates for individuals with high and low DNAm IC (age-adjusted). *P* values were calculated using a log-rank test. **c**, Principal components generated from physical and mental health parameters from 637 individuals. The sample colors represent the DNAm IC estimations. **d**, Significance of the association between health measurements and high or low DNAm IC using logistic regression. Nominal *P* values were derived from two-sided Wald tests. The dot colors and lines indicate the data types in which the variable was measured. The background color for each variable indicates the Pearson’s correlation with age. ECG, electrocardiogram; HDL, high-density lipoprotein. **e**, Correlations between DNAm IC and consumption of different foods, derived flavonoid intake, fatty acid concentration in the blood and dietary adherence. The diamonds indicate significant correlations after correction for multiple testing (FDR < 0.05).

**Extended Data Fig. 6:**
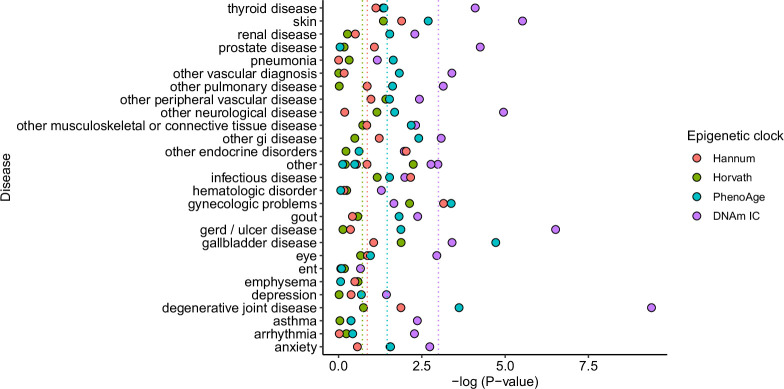
All-cause mortality associations in individuals with diseases. Statistical significance of Cox proportional hazards models for all-cause mortality across different disease subgroups. Dotted lines indicate the mean significance for each epigenetic clock. P-values for the predictor variable were derived from two-sided Wald tests.

Although our IC clock was not trained on mortality data, we hypothesized that the DNAm estimate of IC could also predict mortality, given previous findings showing that IC is a mortality risk factor^17–21^. Using mortality data from the 1,680 individuals in the FHS, we investigated whether DNAm IC was associated with an increased risk of mortality from all causes or age-related conditions. We found that DNAm IC was more strongly associated with all-cause mortality risk than the PhenoAge, Horvath and Hannum clocks (hazard ratio (HR) = 1.38, *P* = 1.67 × 10^−24^) (Fig. 4a). This higher association between DNAm IC and all-cause mortality risk was conserved in individuals in different disease subgroups (Extended Data Fig. 6). In addition, DNAm IC was also more significantly associated with an increased risk of death from age-related diseases, such as cardiovascular disease (HR = 1.29, *P* = 2.37 × 10^−8^), congestive heart failure (HR = 1.33, *P* = 1.62 × 10^−6^) and stroke or TIA (HR = 1.21, *P* = 7.71 × 10^−3^). We examined Kaplan–Meier survival curves for several causes of death, comparing the quintiles with the highest and lowest DNAm IC. Based on the survival curves for all-cause mortality, we estimated that a person with high DNAm IC would live on average 5.5 years longer than someone with low DNAm IC (Fig. 4b).

**Extended Data Fig. 7:**
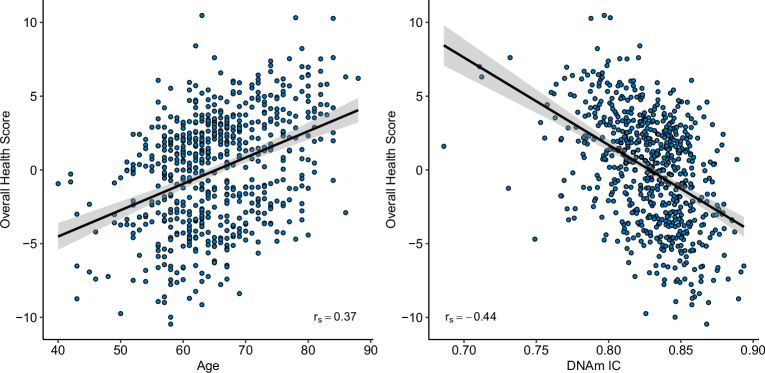
Correlation between health score and chronological age or DNAm IC. Scatterplots depict the relationship between a health score based on clinical data and chronological age (left) or DNAm IC (right) estimates. Correlation estimates were calculated using Spearman’s method (n = 637). Shaded region around the fitted regressions represents the 95% confidence intervals.

We also calculated the association between DNAm IC and assessments of physical and mental health, activities of daily living questionnaires, overall health and biomarkers, and clinical measurements. We first analyzed all markers of health using an integrative approach to derive a healthy aging score. We performed dimensionality reduction (principal component analysis) to extract a single variable representing overall health (that is, principal component 1). We found that overall health was positively correlated with DNAm IC (*r*_*s*_ = −0.44), even more strongly than with chronological age (*r*_*s*_ = −0.37) (Fig. 4c and Extended Data Fig. 7); individuals in the highest and lowest 20% of overall health displayed significant differences in physical and mental health parameters (Supplementary Table 6). When we analyzed individual parameters, we observed that individuals with high DNAm IC displayed better pulmonary function, faster walk time, greater bone mineral density and better self-reported health perception (Fig. 4d and Supplementary Table 7). We also observed a negative association between high DNAm IC and inflammatory age (iAge)^22^, C-reactive protein (CRP), interleukin-6 (IL-6), and markers of neurodegeneration, such as tau and smoking status (Fig. 4d).

Using the comprehensive food frequency questionnaire from the FHS, we explored the relationship between DNAm IC and specific food consumption. We found that individuals with higher DNAm IC consumed more beer (FDR = 5.07 × 10^−6^) and dark meat fish (that is, mackerel, salmon, sardines, bluefish and swordfish) (FDR = 9.39 × 10^–3^) but fewer calcium supplements (FDR = 7.59 × 10^−6^) (Fig. 4e and Supplementary Table 8). The consumption of most flavonoids was associated with higher DNAm IC, but none reached statistical significance after multiple testing correction. Also, elevated blood levels of docosahexaenoic acid (FDR = 9.32 × 10^−10^), docosapentaenoic acid (FDR = 3.71 × 10^−3^) and eicosapentaenoic acid (FDR = 5.24 × 10^−3^), all three long-chain omega-3 fatty acids with marine origin, were associated with higher DNAm IC. Lastly, we analyzed the dietary guideline adherence questionnaire and found that consuming sugar at the recommended level (≤5% of total energy) was associated with a higher DNAm IC (FDR = 3.82 × 10^−2^), suggesting that deviations from the recommended sugar intake guidelines significantly affected functional aging. Overall, these results suggest that consuming fish rich in long-chain omega-3 fatty acids and adhering to the recommended sugar intake guidelines are associated with IC maintenance.

## Discussion

In this study, we constructed a methylation-based clock to monitor age-related decline in IC, which predicts mortality, tracks cardiovascular risk factors and functional resilience, and is strongly associated with immune function and inflammatory health. As we age, both CD4^+^ and CD8^+^ T cells gradually lose their ability to produce CD28, a protein essential for T cell activation and proliferation^23,24^, which ultimately results in immunosenescence and a reduced immune response in older adults^25,26^. Notably, we observed that individuals with high DNAm IC displayed increased expression of *CD28*, higher CD8^+^ naive T cells and lower CD8^+^ exhausted T cells, suggesting that maintaining DNAm IC levels could be an effective strategy for preserving immune function with age.

We found that individuals with high DNAm IC displayed significantly lower iAge, a metric for systemic chronic age-related inflammation^22^. Consistently, we observed negative associations between DNAm IC and the levels of inflammatory markers CRP and IL-6, previously linked with IC^20,27,28^. This could be explained by the loss of CD28 on T cells, which has been associated with increased levels of CRP and inflammatory cytokines such as IL-6 (ref. ^29^).

At the domain-specific level, we observed an interesting association between IC domains and the hallmarks of aging pathways. Examples include vitality with mitochondrial function, psychological with the DNA damage response and sensory with proteostasis. These links between specific aging hallmarks and IC domains help us explain how molecular aging can lead to IC decline and might guide targeted interventions to maintain IC as we age.

We observed that individuals with high DNAm IC displayed improved pulmonary function, which is consistent with the observation that lower levels of IC are associated with an increased risk of respiratory disease mortality^30^. Similarly, individuals with high DNAm IC exhibited improved bone mineral density and engaged in more physical activity, contributing to overall physical resilience^31,32^. Regarding mental capacity, we found that individuals with high DNAm IC had better constructive praxis and reading comprehension in the earliest version of the MMSE^33^. Also, individuals with high DNAm IC had fewer depressive symptoms (Center for Epidemiologic Studies Depression Scale) and lower levels of tau in plasma.

Interestingly, we found that individuals with higher DNAm IC consumed more fish and had elevated levels of marine-origin omega-3 fatty acids in their blood. A recent randomized, controlled trial conducted on 138 sedentary, overweight, middle-aged participants (*n* = 93 women, *n* = 45 men) receiving more than 1 g per day of omega-3 for 4 months, reported positive effects on inflammation and telomere length^34^.

Despite our progress in predicting and understanding the molecular basis of IC, we discovered several areas for improvement. IC rapidly declines at very old age (>90 years old), resulting in few individuals (2.9%) representing half of the potential decline, thereby limiting statistical power to accurately predict very low IC. Also, despite identifying a strong association between IC and the immune system, it is unclear whether causal relationships underlie this association, especially considering that cytomegalovirus infection might potentiate the expansion of CD28^−^CD4^+^ T cells^35,36^.

In summary, we derived a biological clock for age-related decline in IC, which can be estimated from blood and saliva samples and tracks multiple clinical, functional, immune and inflammatory components, as well as lifestyle choices. This biomarker of aging represents a metric for health that can be used to estimate a person’s IC to guide and track aging interventions.

## Methods

### Ethical and regulatory considerations

The INSPIRE-T cohort^37^ is carried out in accordance with the seventh revision of the Declaration of Helsinki (2013), which is the accepted basis for clinical study ethics and must be fully followed and respected by all engaged in research on humans. The INSPIRE-T cohort protocol was approved by the French Ethical Committee in Rennes (CPP Ouest V) in October 2019. This research has been registered with ClinicalTrials.gov (registration NCT04224038). All participants gave written informed consent.

In the FHS cohort, the research protocols are reviewed annually by the Observational Studies Monitoring Board of the National Heart, Lung, and Blood Institute and by the Institutional Review Board of Boston University Medical Center. All participants are required to provide written informed consent before each examination.

### IC score

To calculate the IC score, we used data from the INSPIRE-T cohort (v.1.0). Briefly, the INSPIRE-T cohort is an ongoing 10-year follow-up study investigating IC changes and biomarkers of aging and age-related diseases. Participants were aged from 20 to 102 years; all levels of functional capacity were covered. Assigned sex was obtained from ID cards; no gender information was collected. We calculated an overall IC score based on the following variables describing five health domains: cognition: MMSE (score range = 0–30, higher is better)^33^; locomotion: SPPB (score range = 0–12, higher is better)^38^; psychology: PHQ-9 (score range = 0–27, higher is worse)^39^; sensory: visual acuity measured using the WHO simple eye chart (score range = 0–3, higher is better) and hearing measured using the whisper test (score range = 0–2, higher is better). Although the WHO Integrated Care for Older People Handbook recommends the MMSE, SPPB, PHQ-9, WHO simple eye chart and WHO whisper test as tools to approximate the domains of IC, there is no agreed-upon measure for vitality in the literature. According to the WHO, vitality can include factors related to energy, metabolism, neuromuscular function and the immune response^2^. In this study, we used handgrip strength as a measure of vitality because it is a marker of physiological reserve, which is strongly associated with negative health outcomes, mortality across all ages and disability^40–42^. Handgrip strength also serves as a vital sign for healthy aging throughout the lifespan^43–45^. Additionally, unlike other potential measures of vitality, it is an indicator without fixed minimum and maximum values, thereby limiting the risk of ceiling and floor effects, which is particularly relevant in a lifespan cohort such as INSPIRE-T with participants aged 20–102 years. In contrast to self-reported data, handgrip strength offers an objective, performance-based measure of vitality and is sensitive to age-related changes. It has been extensively used in the literature as a measure of vitality, allowing comparability with previous IC studies^12,46^. From the 1,014 individuals in INSPIRE-T, 973 were assessed in all five domains of IC. Raw scores for each individual were rescaled from 0 to 1, where higher is better (PHQ-9 scores were reversed to match the direction). Given the different score distributions, we *z*-transformed the values. The overall IC score was defined as the average of the *z*-transformed values across domains. We calculated the sensory domain score by averaging the visual and hearing scores. Finally, we performed minimum-maximum normalization on the overall IC score to provide an interpretable metric.

### DNAm data

We performed DNAm profiling (EPIC array) on 1,002 individuals in the INSPIRE-T cohort. We read the raw methylation data using the read.metharray.exp function from minfi v.1.42 (ref. ^47^). We computed the detection *P* values to evaluate sample quality and filtered out samples with a high proportion of failed probes (average *P* > 0.01). We performed initial preprocessing using the preprocessRaw function and identified samples with outlier methylation profiles using QC plots. We removed CpG sites with poor detection *P* values (*P* > 0.01) from any sample. We then converted the methylation dataset into a genomic ratio set and mapped it to the genome. We removed probes at CpG sites with single-nucleotide polymorphisms and cross-reactive probes and excluded sex chromosome probes. We normalized beta values representing methylation levels using the beta-mixture quantile normalization method^48^ implemented in the R package ChAMP v.2.28 (ref. ^49^). We processed DNAm data from the FHS using the same method.

### Model generation

**Extended Data Fig. 8:**
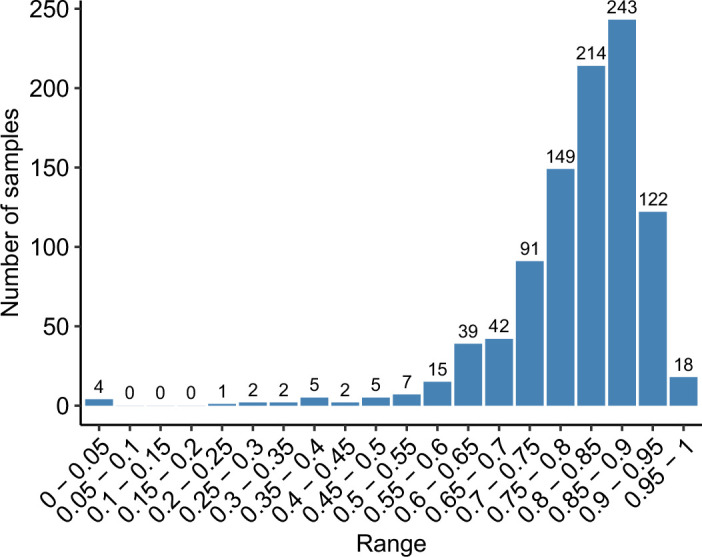
Distribution of IC scores. Number of samples within different ranges of intrinsic capacity.

We used DNAm beta values and IC scores to build a predictive model for IC. To ensure robust predictions, we split the IC score into 20 bins of 0.05 and only considered bins with at least ten individuals for the prediction (Extended Data Fig. 8). This criterion included individuals with an IC between 0.55 and 1. Therefore, we excluded samples with an IC below 0.55, which accounted for 2.9% of the total data. Using the glmnet v.4.1 package^50^, we performed a tenfold cross-validated elastic net regression with alpha parameters ranging from 0.1 to 1, using the mean absolute error as the performance metric. To ensure cross-platform usability, we used data from 361,080 CpGs that passed the quality control tests and overlapped with the 450K array. Using the beta values from the CpGs, the elastic net regression algorithm selected sets of CpG sites at different levels of feature sparsity, with stronger penalties (for example, alpha = 1) selecting fewer features and weaker penalties (for example, alpha = 0.1) including more features. The best-performing model based on the lowest mean absolute error, highest correlation with IC and the smallest number of features, used an alpha of 0.9 and included 91 CpG sites. To facilitate calculation of the IC clock, in addition to the inclusion of the model coefficients as supplementary material, we developed a simple web application (https://mfuentealba.shinyapps.io/icclock/) that allows users to calculate the DNAm IC from an uploaded file containing DNAm beta values (that is, CpG identifier as the first column and sample beta values are the remaining columns).

### Comparisons with epigenetic clocks

We compared our IC clock with established epigenetic clocks using IC predictions based on DNAm (DNAm IC) and epigenetic age estimates calculated with the methylclock package v.1.2.1 (ref. ^51^), including the Horvath, Hannum and Levine clocks. We computed age acceleration as the residuals from the linear model between chronological age and DNAm age or DNAm IC. We calculated Spearman’s rank correlation coefficients to evaluate the relationships between the IC clock and each epigenetic clock.

### Gene expression enrichment

Using the generated model, we predicted IC for individuals in the Offspring cohort of the FHS using DNAm data (obtained during the eighth exam) and investigated its association with gene expression changes, followed by enrichment analysis. We performed differential gene expression analysis using linear models to identify genes associated with age-adjusted DNAm IC. We filtered genes with an FDR < 0.05. We conducted enrichment analysis using the clusterProfiler package v.4.6.2 (ref. ^52^) to identify the biological processes associated with differentially expressed genes. We visualized enriched GO terms using the CellPlot package v.1.0 (https://github.com/dieterich-lab/CellPlot).

### Hallmark of aging enrichment

To identify the genes linked to the 12 hallmarks of aging, we used a corpus of 36 million abstracts from PubMed (https://huggingface.co/datasets/ncbi/pubmed). First, we identified 71,129 abstracts that included the word ‘aging’ or ‘ageing’ in the title or abstract. Then, we used large language models (GPT-4o mini) to analyze each abstract using the following query: ‘Your task is to identify genes associated with the hallmarks of aging from the following scientific abstract. For each gene mentioned in the abstract, annotate it with the corresponding hallmark of aging (genomic instability, telomere attrition, epigenetic alterations, loss of proteostasis, deregulated nutrient sensing, mitochondrial dysfunction, cellular senescence, stem cell exhaustion, altered intercellular communication, disabled autophagy, chronic inflammation and dysbiosis)’. To perform enrichment analysis using these gene sets, we ranked the genes in the query signature according to −log10(*P*) and then performed a one-signed gene set enrichment analysis using the package fgsea v.1.27 (ref. ^53^).

### Cell count estimation

We estimated the proportions of CD8^+^ T cells, CD4^+^ T cells, natural killer (NK) cells, B cells and granulocytes using Houseman’s estimation via the meffilEstimateCellCountsFromBetas function implemented in the methylclock package v.1.2.1. We also estimated the number of CD8^+^ naive and CD8^+^ cytotoxic T cells, and plasmablasts, using the blood cell count predictors developed in ref. ^54^. We integrated the age data and calculated correlations between cell counts and age using Spearman’s rank correlation coefficients. We also conducted correlation analyses between age-adjusted cell counts and age-adjusted DNAm IC.

### Mortality analysis

We compared DNAm-based IC (DNAm IC) and traditional epigenetic clocks (Horvath, Hannum and PhenoAge) for their ability to predict mortality using the FHS data. We computed age-adjusted and sex-adjusted residuals for DNAm IC and each clock. We grouped participants into quintiles based on the DNAm IC residuals, comparing the top and bottom 20%. Using the R package survival v.3.5.7, we performed Cox proportional hazards models^55^, adjusted for age and sex, to calculate the HRs and 95% CIs associated with mortality from all causes, cardiovascular disease, congestive heart failure and stroke/transient ischemic attack (TIA). We plotted Kaplan–Meier survival curves to visualize survival differences between high and low IC groups, and calculated *P* values using a log-rank test^56^.

### Association with health-related assessments and lifestyle

We analyzed the correlation between age-adjusted IC (top and bottom quintiles) and several physiological and clinical measurements using data from the FHS, including clinic laboratory assays, inflammatory markers, tau levels, cardiovascular risk factors and dietary data using logistic regression. We calculated Spearman’s rank correlation coefficients and *P* values for the relationship between IC and each feature, applying FDR adjustments for multiple comparisons. Significant correlations (FDR < 0.05) were visualized using the ComplexHeatmap package v.2.12 (ref. ^57^); scatter plots were created for dietary factors (flavonoids, fatty acids, food frequency and dietary guideline adherence).

All the analyses were performed using R v.4.2.3, run on the RStudio Server v.2022.02.3 build 492. Data handling and visualization also included the use of the following R packages: tidyverse v.2.0; ggpubr v.0.4; RColorBrewer v.1.1.3; circlize v.0.4.15; khroma v.1.10; gridExtra v.2.3; ggrepel v.0.9.1; viridis v.0.6.2; ggplot2 v.3.5.1; ggh4x v.0.2.8.9; and ggsci v.2.9.

### Statistics and reproducibility

No data were excluded from generating the IC scores in INSPIRE-T, except for samples that did not measure all relevant IC domains. For model training, we excluded individuals with low IC scores from bins (0.05 increments) containing fewer than ten participants to ensure robust predictions. For the FHS cohort validation analysis, we only excluded samples whose DNAm data did not pass quality control.

### Reporting summary

Further information on research design is available in the Nature Portfolio Reporting Summary linked to this article.

## Supplementary information

Reporting Summary

Supplementary Tables 1–8.

## Data Availability

The de‑identified data for the INSPIRE‑T cohort are available under controlled access because of privacy, ethical and legal requirements. Researchers requiring access should contact the INSPIRE Data Access Committee (guyonnet.s@chu‑toulouse.fr) and submit a research proposal for approval. Access will be granted once the proposal is approved and a data use agreement has been signed. The data used to validate the study observations come from the FHS cohort, which requires a data access request via database of Genotypes and Phenotypes (www.ncbi.nlm.nih.gov/projects/gap/cgi-bin/study.cgi?study_id=phs000007). Controlled access is required to protect participant privacy and comply with ethical and legal requirements. Researchers must submit a research proposal along with institutional review board approval documentation. Access will be granted after review by the database of Genotypes and Phenotypes Data Access Committee and execution of a data use certification agreement cosigned by a designated institutional signing official.

## Code availability

All custom code used for the analyses is available on GitHub at https://github.com/msfuentealba/IC_clock.
