# Supplementary material for: A blood-based epigenetic clock for intrinsic capacity predicts mortality and is associated with clinical, immunological and lifestyle factors
Source: Nat Aging. 2025 Jun 4;5(7):1207–16. doi: 10.1038/s43587-025-00883-5 (PMC12270914; doi:10.1038/s43587-025-00883-5)
Supplement: Supplementary file 1 — Reporting Summary [file 43587_2025_883_MOESM1_ESM.pdf]

nature portfolio

Corresponding author(s): David Furman
Last updated by author(s): Apr 7, 2025

Reporting Summary

Nature Portfolio wishes to improve the reproducibility of the work that we publish. This form provides structure for consistency and transparency in reporting. For further information on Nature Portfolio policies, see our Editorial Policies and the Editorial Policy Checklist.

Statistics

For all statistical analyses, confirm that the following items are present in the figure legend, table legend, main text, or Methods section.

n/a	Confirmed	
☐	☒	The exact sample size (*n*) for each experimental group/condition, given as a discrete number and unit of measurement
☐	☒	A statement on whether measurements were taken from distinct samples or whether the same sample was measured repeatedly
☐	☒	The statistical test(s) used AND whether they are one- or two-sided<br>*Only common tests should be described solely by name; describe more complex techniques in the Methods section.*
☐	☒	A description of all covariates tested
☐	☒	A description of any assumptions or corrections, such as tests of normality and adjustment for multiple comparisons
☐	☒	A full description of the statistical parameters including central tendency (e.g. means) or other basic estimates (e.g. regression coefficient) AND variation (e.g. standard deviation) or associated estimates of uncertainty (e.g. confidence intervals)
☐	☒	For null hypothesis testing, the test statistic (e.g. *F*, *t*, *r*) with confidence intervals, effect sizes, degrees of freedom and *P* value noted<br>*Give P values as exact values whenever suitable.*
☒	☐	For Bayesian analysis, information on the choice of priors and Markov chain Monte Carlo settings
☒	☐	For hierarchical and complex designs, identification of the appropriate level for tests and full reporting of outcomes
☐	☒	Estimates of effect sizes (e.g. Cohen's *d*, Pearson's *r*), indicating how they were calculated

Our web collection on statistics for biologists contains articles on many of the points above.

Software and code

Policy information about availability of computer code

Data collection	The primary data for this study come from the INSPIRE-T and Framingham Heart Study cohorts. We did not collect additional data for INSPIRE-T or Framingham Heart Study. We used R v4.2.3 to access other publicly available, programmatically accessible data resources.
Data analysis	Analyses were performed using R v4.2.3, run on RStudio server v2022.02.3 build 492. The following packages were used: tidyverse v2.0, ggpubr v0.4, RColorBrewer v1.1.3, circlize v0.4.15, khroma v1.10, gridExtra v2.3, ggrepel v0.9.1, viridis v0.6.2, ggplot2 v3.5.1, ggh4x v0.2.8.9, ggsci v2.9, ComplexHeatmap v2.12, CellPlot v1.0, clusterProfiler v4.6.2, fgsea v1.27, methylclock v1.2.1, survival v3.5.7, ChAMP v2.28, minfi v1.42, glmnet v4.1. All analyses were performed using custom code written in R and are available on Github: https://github.com/msfuentealba/IC_clock.

For manuscripts utilizing custom algorithms or software that are central to the research but not yet described in published literature, software must be made available to editors and reviewers. We strongly encourage code deposition in a community repository (e.g. GitHub). See the Nature Portfolio guidelines for submitting code & software for further information.

Data

Policy information about availability of data

All manuscripts must include a data availability statement. This statement should provide the following information, where applicable:

- Accession codes, unique identifiers, or web links for publicly available datasets
- A description of any restrictions on data availability
- For clinical datasets or third party data, please ensure that the statement adheres to our policy

The INSPIRE-T cohort data is accessible via direct request (https://ihuhealthage.fr/en/human-cohort/). The data used to validate the study observations come from the Framingham Heart Study cohort which requires an application for access via dbGaP (https://www.ncbi.nlm.nih.gov/projects/gap/cgi-bin/study.cgi?study_id=phs000007.v34.p15). The model generated during the study to estimate intrinsic capacity using DNA methylation data is available at https://mfuentealba.shinyapps.io/icclock/ and supplementary table 1.

Research involving human participants, their data, or biological material

Policy information about studies with human participants or human data. See also policy information about sex, gender (identity/presentation), and sexual orientation and race, ethnicity and racism.

Reporting on sex and gender	The findings of this study apply to both sexes, as intrinsic capacity was assessed in both males and females. Sex was considered in the study design and analyses were conducted to evaluate sex differences in intrinsic capacity domains. Sex was assigned based on identity cards. Gender was not collected or analyzed in INSPIRE-T.
Reporting on race, ethnicity, or other socially relevant groupings	The study did not include analyses of the differences in race and ethnicity because of under-representation of comparable racial and ethnic groups in the training and validation cohorts.
Population characteristics	The study population of INSPIRE-T consists of 1,068 males and females aged 20 to 102 years of age with varying levels of functional status at baseline (robust, pre-frail, frail). Eligibility criteria excluded individuals with severe diseases compromising life expectancy at 5 years (or at 2 years for frail older adults and those aged 80 years and older) and individuals who were legally incapable. Participants were stratified into ten-year age groups, with oversampling of people older than 70 years.
Recruitment	Participants in INSPIRE-T were recruited from the Toulouse region of France. INSPIRE-T participants were recruited from several sources. The largest proportion of participants (30.0%) was recruited through media outlets, followed by personal contact (24.1%). Hospital outpatient clinics accounted for 14.7%, and online promotion contributed 9.8%. Smaller proportions were recruited from previous studies or existing registries (11.6%), public conferences, and residential homes or nursing homes (5.6%).
Ethics oversight	The INSPIRE-T cohort is conducted in accordance with the Declaration of Helsinki, which is the accepted basis for clinical study ethics, and must be fully followed by all engaged in research on human beings. The INSPIRE-T cohort protocol was approved by the French Ethical Committee located in Rennes (CPP Ouest V) on October 2019. The INSPIRE-T cohort study was registered at http://clinicaltrials.gov (ID NCT04224038). All participants gave written informed consent.

Note that full information on the approval of the study protocol must also be provided in the manuscript.

Field-specific reporting

Please select the one below that is the best fit for your research. If you are not sure, read the appropriate sections before making your selection.

☒ Life sciences ☐ Behavioural & social sciences ☐ Ecological, evolutionary & environmental sciences

For a reference copy of the document with all sections, see nature.com/documents/nr-reporting-summary-flat.pdf

Life sciences study design

All studies must disclose on these points even when the disclosure is negative.

Sample size	Sample size calculation was not relevant as many objectives of the INSPIRE-T cohort are exploratory. We therefore considered an approach based more on the potential of the INSPIRE-T cohort in terms of the ability to obtain parameter estimates with sufficient precision with a recruitment of 1000 subjects that corresponds to the maximum number of subjects that can be recruited and monitored with the funding provided. In case of evident underpowered population (for a particular subgroup of subjects), a reasoned additional recruitment of subjects may be considered in a second phase. To limit the attrition rate, subjects will be monitored by both active (visits, telephone calls) and passive ways (monitoring of several functions using new technologies via mobile phones or other connected devices).
Data exclusions	No data were excluded from generating the IC scores in INSPIRE-T, except for samples that did not measure all relevant IC domains. During the generation of the DNA methylation predictor of intrinsic capacity, 2.9% of participants in the INSPIRE-T cohort with epigenetic data available were excluded due of unusually low intrinsic capacity scores. While these might be biologically relevant observation, the undersampling of this population did not allow building accurate models to predict intrinsic capacity. For the FHS cohort validation analysis, we only excluded samples whose DNA methylation data did not pass quality control.

Replication	A 10-fold cross-validation was used in the analysis to ensure that the predictive model for intrinsic capacity based on DNA methylation was robust and reproducible. The findings from the INSPIRE-T cohort were further replicated using the Framingham Heart Study as an external dataset to validate the associations between DNA methylation and intrinsic capacity.
Randomization	The current study did not include randomization. All analyses included all available samples, except in cases of poor sample quality or when a variable was not measured. All analyses, including DNAm IC, included age as a covariate.
Blinding	The study did not involve random treatment allocation or require blinding. Its primary focus was to analyze the associations between intrinsic capacity and clinical data across a diverse population.

Reporting for specific materials, systems and methods

We require information from authors about some types of materials, experimental systems and methods used in many studies. Here, indicate whether each material, system or method listed is relevant to your study. If you are not sure if a list item applies to your research, read the appropriate section before selecting a response.

Materials & experimental systems

n/a	Involved in the study
☒	☐ Antibodies
☒	☐ Eukaryotic cell lines
☒	☐ Palaeontology and archaeology
☒	☐ Animals and other organisms
☒	☐ Clinical data
☒	☐ Dual use research of concern
☒	☐ Plants

Methods

n/a	Involved in the study
☒	☐ ChIP-seq
☒	☐ Flow cytometry
☒	☐ MRI-based neuroimaging

Plants

Seed stocks	n/a
Novel plant genotypes	n/a
Authentication	n/a
